# Facile Functionalization via Plasma-Enhanced Chemical Vapor Deposition for the Effective Filtration of Oily Aerosol

**DOI:** 10.3390/polym11091490

**Published:** 2019-09-12

**Authors:** Sanghyun Roh, Sungmin Kim, Jooyoun Kim

**Affiliations:** 1 Department of Textiles, Merchandising and Fashion Design, Seoul National University, Seoul 08826, Korea; 2 Research Institute of Human Ecology, Seoul National University, Seoul 08826, Korea

**Keywords:** plasma, filtration, loading, surface energy, charges

## Abstract

With the growing concern about the health impacts associated with airborne particles, there is a pressing need to design an effective filter device. The objective of this study is to investigate the effect of plasma-based surface modifications on static charges of electrospun filter media and their resulting filtration performance. Polystyrene (PS) electrospun web (ES) had inherent static charges of ~3.7 kV due to its electric field-driven process, displaying effective filtration performance. When oxygen species were created on the surface by the oxygen plasma process, static charges of electret media decreased, deteriorating the filter performance. When the web surface was fluorinated by the plasma-enhanced chemical vapor deposition (PECVD), the filtration efficiency against oily aerosol significantly increased due to the combined effect of decreased wettability and strong static charges (~−3.9 kV). Solid particles on the charged media formed dendrites as particles were attracted to other layers of particles, building up a pressure drop. The PECVD process is suggested as a facile functionalization method for effective filter design, particularly for capturing oily aerosol.

## 1. Introduction

The World Health Organization (WHO) has reported that the ambient air pollution accounts for an annual death of about 4.2 million in 2016 by various chronic respiratory diseases [1]. Especially the Southeast Asia region, such as China and Korea, suffers a lot from the severe pollution caused by the airborne particles that consist of a mixture of fine dust, liquid mist, volatile organic compounds and possibly microbials [2,3,4,5]. The health risks associated with the airborne particles become serious for the particle size less than 2.5 µm, as such fine matters can even reach the bloodstream [3]. With such concerns, the role of air filters either in the form of air-purifying respirators or a filtration system become more important.

Particles in the airflow can be filtered through a fibrous media by the single fiber-capture mechanism, not by sieving [3,6,7]. Thus, a filter media with large open pores can capture particles smaller than its pore size. Among the capture mechanisms, including interception, inertial impaction, diffusion, etc., the electrostatic attraction is most helpful in capturing particles of the most penetrating particle size (MPPS) in the diameter range of about 0.1–0.5 µm [6,7,8]. Thus, electrostatic charges are often imposed upon the filter media during manufacturing to better attract: (1) The oppositely-charged particles by the coulombic interaction, and (2) the neutral particles by induced polarization [9,10,11]. While the capture efficiency of different sized-particles varies depending on the air velocity, very fine particles smaller than MPPS are captured rather efficiently by the diffusion mechanism [12]. 

To reflect the worst case scenario, most of regulatory standards for testing air-filtering respirators use the particles in the MPPS range; for example, the US National Institute for Occupational Safety and Health (NIOSH) tests with neutralized NaCl solid particles with the count median diameter (CMD) of ~0.075 µm and dioctyl phthalate (DOP) with a CMD of ~0.185 µm [6,13].

The development of filter media has been driven to give a high filtration efficiency at the low air resistance (or pressure drop) during the mass loading [14,15,16,17,18,19,20]. The resistance is an important parameter for respirators, as a low resistance is translated as better breathing comfort to users. To account for both efficiency and pressure drop, the concept of a quality factor (QF), the relative capture efficiency to the resistance, is commonly employed, where a higher QF indicates a higher efficiency at the same pressure drop. To design a filter with a high QF, it is desirable for fibers to have a high specific area and tortuous air pathways, as it would increase the probability of particles’ contact to fibers [21]. 

Most of the commercially-available, electrostatically-charged (electret) filters are made of polypropylene (PP) meltblown web, often with a separate step of the charging process. PP is relevant for filter application due to: (1) Its facile meltblown processability, and (2) its hydrophobicity that allows the imposed charges to sustain longer in a humid environment [9,22,23]. Recently, electrospinning is recognized as a way to fabricate effective filter materials, due to its high specific area with submicron fibers, and the inherent charges resulting from the electric field-driven process [8,16,17,21,24,25,26,27,28,29,30,31,32,33]. However, nanoscale fibers with a high packing density may result in a high air resistance that leads to an earlier clogging and shorter service life [34,35,36]. Thus, to apply to a filter material, the electrospinning process needs to be carefully adjusted to optimize the efficiency and the resistance throughout the particle loading. 

Generally, the filter efficiency is deteriorated more easily by the oily aerosol than the solid particles, because the oily liquid spreads on fiber surface, quickly masking the charged sites. To enhance the performance against the oily aerosol by deterring the immediate wetting of the oily liquid, an anti-wetting surface treatment can be applied to the electret media. While it is speculated that the static charges on electrospun fibers would help filtration, the effect of inherent charges and anti-wetting treatment on filtration performance has rarely been investigated. 

**Figure 1:**
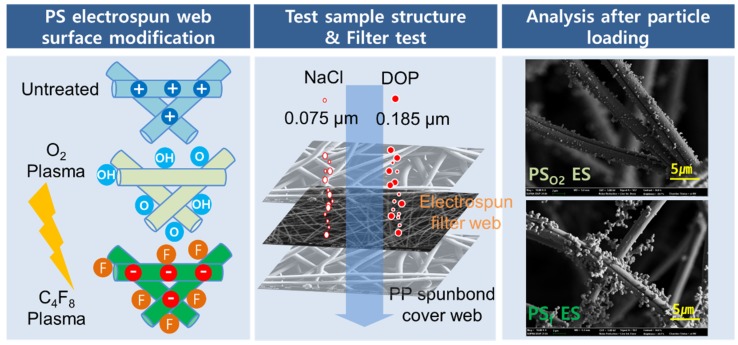
Schematic overview of this study.

The specific aim of this study is to investigate the effect of plasma-based surface functionalization for electrospun fibers on the static charges and the filtration performance. As the filter media, electrospun webs made of polystyrene (PS), with and without surface modification, were employed. The surface functionality of the PS web was modified by the plasma processes [37,38,39,40,41]. To attach hydrophilic oxygen species onto a PS surface, O_2_ plasma was treated to the web [42]. For surface fluorination, the plasma-enhanced chemical vapor deposition (PECVD) with C_4_F_8_ (in the form of octafluorocyclobutane) was conducted. As a measure of filter performance, evolvement of particle penetration and air resistance was examined during the loading of NaCl solid particles and DOP oily aerosol. The filtration performance of the electrospun web (ES) was compared to that of PP meltblown (MB), which is readily available for commercial products. The surface chemistry of the filter media was examined using x-ray photon spectrometry (XPS) to discuss the effect of chemical functionality on static charges and filtration performance. The ultimate goal of this study is to provide design insights for effective filter media that have superior filtration performance and enhanced service life. The schematic overview of this study is illustrated in Figure 1.

## 2. Materials and Methods 

### 2.1. Materials

Polystyrene (PS) pellets (M_w_ ~350,000) were purchased from Sigma Aldrich (St. Louis, MO, USA). *N*,*N*-dimethylformamide (DMF), and tetrahydrofuran (THF) were purchased from Fisher Scientific (Hampton, NH, USA). Spunbond polypropylene (PP) was provided by the Gaurdman (Incheon, Korea), and meltblown electret filter media was provided by the Korea Institute of Industrial Technology (KITECH) (Cheonan, Korea). PP film was made by casting the PP resin obtained from SK Global Chemical Co., Ltd. (Seoul, Korea), and PS films were purchased from Goodfellow (Huntingdon, UK). Sodium chloride (ACS grade) and dioctyl phthalate (DOP, ACS grade) were purchased from the Showa Chemical Industry Co., Ltd (Meguro-ku, Tokyo, Japan) and Junsei Chemical Co., Ltd. (Chuo-ku, Tokyo, Japan), respectively. Methylene iodide (99.0%) was purchased from Alfa Aesar (Haverhill, MA, USA). Octafluorocyclobutane (C_4_F_8_) gas and oxygen gas (O_2_) were purchased from Union Gas (Yongin, Korea).

### 2.2. Preparation of PS Electrospun Filter Media

The electrospun web with a basis weight of about 14 g/m^2^ and thickness of 0.13 mm was prepared as following: The PS resin was dissolved in a solvent mixture of DMF and THF in a 1:3 *v*/*v* ratio to make a 20% (*w*/*v*) pre-spinning solution. An electrospinning apparatus (ESR200D, NanoNC, Seoul, Korea) was set to feed the PS pre-spinning solution at 6 mL/h at 12 kV of applied voltage, and the electrospun fibers were collected on a drum collector rotating at 100 rpm. The needle gauge of 23 (O.D. 0.64 mm, I.D. 0.34 mm) was used as an electrospinning tip. The tip to collector distance was set as 14 cm. The chamber temperature and relative humidity were 15 ± 5 °C and 30 ± 5% RH, respectively. All electrospun samples were used only after drying for at least for 24 h at ambient condition to completely evaporate the solvent. 

The surface chemistry of the electrospun web was modified. To attach the oxygen group to the PS surface, the web was subjected to an O_2_ plasma treatment for 5 min at 200 W with 160 sccm in the plasma system (COVANCE, FemtoScience, Hwaseong, Korea) [43,44,45]. To coat the surface with the fluorinated compound by the PECVD process, the PS web was treated under C_4_F_8_ gas for 25 min at 200 W with 100 sccm [44]. The generated frequency of the plasma was 50 kHz in both the O_2_ and C_4_F_8_ plasma processes.

### 2.3. Characterization

The atomic percentage of different nonwoven media was analyzed in tens of nanometers depth using an x-ray photoelectron spectrometer (XPS, Axis SupraTM, Kratos Analytical, Manchester, UK), to examine the effect of plasma treatment on surface chemistry of the fibrous media. The surface morphology was observed using a field-emission scanning electron microscope (FE-SEM, Supra 55VP, Carl Zeiss, Jena, Germany), with prior Pt coating (~10 nm) at 30 mA for 200 s using a sputter coater (EM ACE200, Leica, Wetzlar, Germany). 

The wettability of the web surface was examined via the measurement of static contact angles (CA) and shedding angles (ShA), using an optical tensiometer (Theta Lite, KSV Instruments Ltd., Espoo, Finland). For contact angle (CA) measurement, a 3.4 µL of liquid drop was placed upon a surface, and the CA was measured within 5 s after deposition of this liquid drop. The measurement was done on at least five different locations of the sample surface. For the shedding angle measurement, a 12.5 µL liquid was dropped vertically from a 1 cm distance above the sample surface that was on a tilted stage. The lowest tilting angle at which the drop starts to roll more than 2 cm upon the surface was recorded as the shedding angle, and this process was repeated at least five times. 

The surface energies of PS surfaces, untreated and treated, were estimated by measuring CAs of water (WA) and methylene iodide (MI) on flat film surfaces, and employing the Owens-Wendt model [46]. The calculation for the surface energy, and its dispersive and polar components, is shown in the supplementary information (Table S1 and Eqations (S1)–(S4) in Supplementary Materials). The surface energy of the PS web was regarded as the same as that of the PS film. For PP surfaces (PP meltblown and PP spunbond), the same procedure was applied using a PP flat film. 

The static charges of the webs were measured using a handheld electrostatic fieldmeter (DS-MFX-004, SIMCO IONTM, Alameda, CA, USA). The web samples in 8 cm × 8 cm were hung in the air, and static charges from nine different locations were measured, keeping the distance 2.54 cm (one inch) away from the surface. 

Pore size distribution of the web was measured using the capillary flow porometer (CFP 1500AE, PMI Inc., Ithaca, NY, USA). Effective fiber diameters (EFD) of the test samples were calculated as a relative fiber size, as in Eqation (1) [47]. The solidity and porosity of the test samples were calculated as Eqations (2) and (3), respectively [25].

(1) $$\mathrm{EFD}\;\left(µm\right)=10^{4}\sqrt{\frac{{\eta \;\mathrm{tU}}_{0}{64\alpha }^{1.5}{(1+56\alpha }^{3})}{\mathrm{dP}}}$$

Solidity (α) = m/(A⋅t⋅ρ)(2)

Porosity (%) = [1 − solidity] × 100 (%)(3)

α (unitless): Solidity or solids fraction of the material; dP (mmH_2_O): Air flow resistance; pressure drop, η (g⋅s/cm): Air viscosity [48], 1.81 × 10^−4^ g·s/cm at 20 °C; t (mm): Sample thickness; U_0_ (cm/s): Face velocity (11.8 cm/s in this study); m (g): Sample mass; A (${\mathrm{cm}}^{2}$): Sample area; ρ (g/${\mathrm{cm}}^{3}$): Polymer density (1.04 g/cm^3^ for PS, 0.91 g/cm^3^ for PP).

### 2.4. Filtration Performance

**Figure 2:**
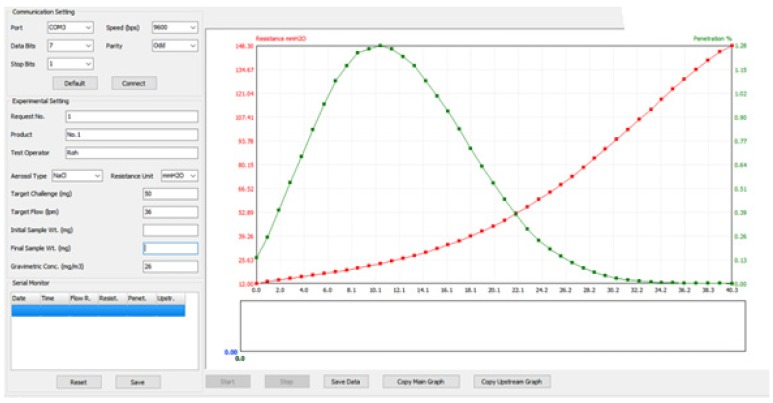
Data acquisition program developed in-house.

For filter testing, a layer of electrospun test sample was layered with PP SBs at the top and the bottom each, to prevent the tearing of our test sample during the test. An automated filter tester (TSI 8130, TSI Inc., Shoreview, MN, USA) was used to evaluate the particle penetration (%) and resistance (mmH_2_O) during the mass loading of challenging agents. The in-house data acquisition system was developed to monitor penetration, resistance, flow rate and the challenged amount of particulate matters. The operating software is captured in Figure 2.

As challenging agents, DOP oily aerosol (CMD ~0.185 ± 0.02 µm) and NaCl solid particles (CMD ~0.075 ± 0.02 µm), with their equilibrium charges neutralized, were used. A sample area of 40 cm^2^ was exposed to the airflow with NaCl (18.6 ± 0.9 g/L) or DOP (67.8 ± 4.0 g/L) at the flow rate of 28.3 l/min, which corresponds to ~11.8 cm/s of face velocity. 

The loading of the challenge agent was carried out until 40 mg of NaCl or 200 mg of DOP was challenged onto the test samples. The % penetration of NaCl and DOP through the test sample was determined by reading the relative concentration in the upstream and downstream of the test sample (Equation (4)). The quality factor (QF), the relative filtration efficiency to the pressure drop, was calculated as Equation (5) to compare the filtration performance of media with different basis weight and thickness [49].

(4) $$\mathrm{Penetration}\;(\%)=\frac{\mathrm{Downstream}\;\mathrm{concentration}}{\mathrm{Upstream}\;\mathrm{concentration}}\text{×}100\%$$

(5) $$\mathrm{Quality}\;\mathrm{factor}\;({\mathrm{mmH}}_{2}O^{-1})=-\ln\left(\frac{\%\;\mathrm{penetration}/100\%}{\mathrm{resistance}\;\left({\mathrm{mm}\;H}_{2}O\right)}\right)$$

The data of filtration efficiency measured by filter tester TSI 8130 was recorded with an independently developed recording program (Figure 2).

## 3. Results

### 3.1. Fabrication of Electrospun Media with Different Functionalities

In manufacturing electret filter media, the charging process is commonly applied as a separate step, and the imposed charges hardly dissipate in a normal environment, as an insulating polymer is generally used as the filter material. The electrospinning process is advantageous for fabricating the filter media, as the static charges can be immediately imposed onto the produced fibers, without undergoing an additional charging step. PS electrospun fibers can form various morphologies and sizes, depending on the choice of solvents and process conditions. As a compact structure with nano-sized fibers can lead to a high air resistance and earlier clogging, the electrospinning condition was adjusted to produce the microscale fibers in this study (Table 1). It should be noted that the calculated EFD does not exactly match with the actual fiber diameters measured from the SEM images. The porosity of all tested webs were ranged in 89–90% (Table S2 in Supplementary Materials). The overall web characteristics are presented in Table 1.

According to the NIOSH standards of R- and P-type particulate respirators, filter media need to maintain the high efficiency during 200 mg of DOP loading. As the surface charges of electret media can be quickly masked by the oil spreading on a fibrous surface, the anti-wetting treatment of the fiber surface can be beneficial for keeping the performance of R- and P-type filters. For an anti-wetting chemical treatment on a non-reactive surface such as PS, the plasma-enhanced chemical vapor deposition (PECVD) can be useful, yet the effect of this PECVD process on static charges and the resulting filtration performance is a question. In addition, the effect of the plasma-based hydrophilic surface treatment on the static charges and the filtration have not been previously examined. To investigate the influence of surface functionality on charges and filtration, the PS surface was treated for C_4_F_8_ PECVD (PS_f_) and O_2_ plasma (PS_O_2__). PS polymer itself is a hydrophobic polymer with low surface energy, thus the water (WA) contact angle (CA) was not changed much when the surface was fluorinated. The shedding angle (ShA) measurement is usually a better discriminator than the CA for the hydrophobic surfaces [43,44]; From Table 2, the ShA of PS_f_ electrospun web (ES) is lower (11°) than that of PS ES (41°), clearly demonstrating the enhanced anti-wetting property of the fluorinated PS (PS_f_ ES) by the PECVD process. When PS ES was treated by O_2_ plasma, the wettability of PS_O_2__ was significantly increased, as shown by a WA CA of 0° (immediate wetting) [42]. PP MB had high WA CA (155°) and a low ShA (14°) due to its intrinsic hydrophobicity. PP SB showed lower ShA than PP MB, due to the spot-bonding points that deterred the rolling of the water droplet. Nonetheless, the WA CA on PP SB was as high as 154°. As the webs were tested against the DOP aerosol, the wettability of DOP was also presented in Table 2. The oil wetting was significantly lowered for the fluorinated surface (PS_f_ ES), while all other surfaces displayed complete wetting of DOP (CA of 0°).

The surface chemistry with and without plasma treatments were examined by the atomic concentration (%) of X-ray photoelectron spectroscopy (XPS) analysis. As the fine fibers which protruded from the web inhibited the XPS analysis, the film surfaces treated by the same plasma processes, instead of web surfaces, were analyzed for XPS. The result in Table 3 demonstrates the effective modification of the surface chemistry by the plasma processes; it confirmed that the modified wettability of the PS_O_2__ and PS_f_ surface is the result of the presence of hydrophilic oxygen species and hydrophobic fluorinated compounds on the treated surfaces, respectively. 

**Figure 3:**
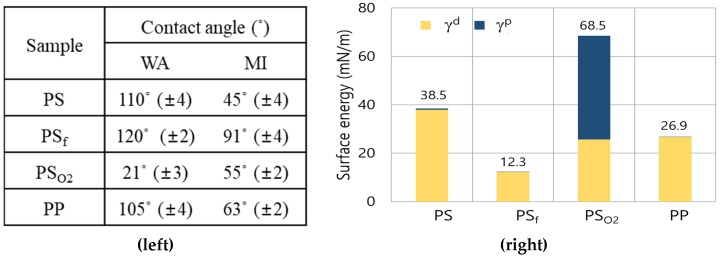
Surface energy of materials. (**Left**), contact angle measurements of water (WA) and methylene iodide (MI); (**Right**), surface energy components of materials. γ, total surface energy; γ^d^ and γ^p^, dispersive and polar components of surface energy, respectively. For WA, the dispersive and polar components of 21.8 and 51.0 mN/m, respectively, were used [50]. For MI, the dispersive and polar components of 50.4 and 0.4 mN/m, respectively, were used [50].

In Figure 3, the surface energy of different surfaces was calculated by measuring the CAs of WA and methylene iodide (MI) from the flat film surfaces (Figure 3, Left) and employing the Owens-Wendt model [43,46]. The PS_O_2__ surface increased the polar component of the surface energy (γ^p^) due to the oxygen groups on the surfaces, and the PS_f_ surface significantly decreased the total surface energy (γ). PP also showed a low γ; as the PP is fully composed of hydrocarbons, then the γ^p^ is close to zero. While γ^p^ of PS was slightly higher than that of PP, γ of PS_f_ was lower than that of PP. The information given in Table 2 and Table 3, Figure 3 demonstrates that the plasma treatments effectively modified the surface energy and the chemistry of materials, affecting the liquid wettability. 

### 3.2. Filtration Performance

The filtration performance of PS webs with and without plasma treatments were examined, comparing with a reference web of PP MB electret. As the ES and MB webs were flimsy and weak, a single layer of the ES or MB test sample was layered between the PP SBs on the top and the bottom. Since the PP SB web showed the negligible efficiency (<2% for DOP; <5% for NaCl) and the resistance (<0.2 mm H_2_O), the added performance by the PP SB layers was disregarded in the later analysis. The NIOSH standard requires us to record the maximum penetration during 200 mg of particle loading. As DOP penetration keeps increasing until 200 mg of loading, the maximum penetration was observed until 200 mg of DOP aerosol was challenged. For NaCl, the maximum penetration appeared earlier than 40 mg of loading, and thereafter this penetration decreased due to a clogging of the filter media. Therefore, the NaCl test was stopped at 40 mg mass loading, and the maximum penetration was recorded. 

**Figure 4:**
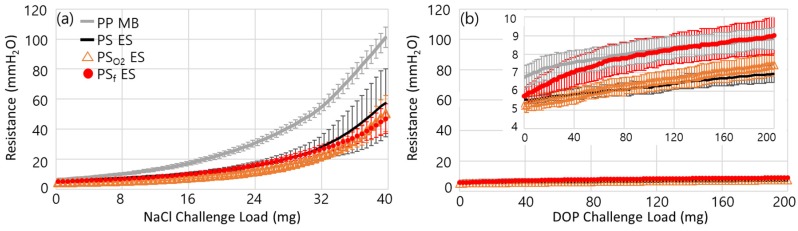
Evolvement of air resistance during the loading of challenge agents. (**a**) Resistance at NaCl loading; (**b**) Resistance at DOP loading.

**Figure 5:**
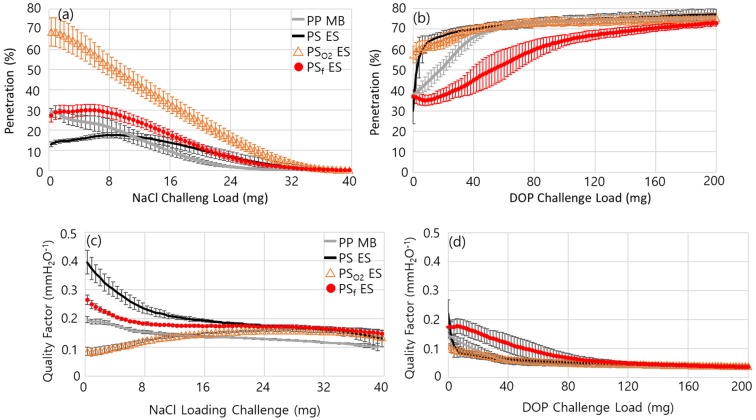
Evolvement of penetration and quality factor (QF) during particle loading. (**a**) NaCl penetration; (**b**) DOP penetration; (**c**) QF with NaCl loading; (**d**) QF with DOP loading.

Figure 4 shows the evolvement of air resistance during the particle loading. In general, solid particles such as NaCl are captured by the fiber, forming dendrites; such particles easily block the pores of the web, and the resistance develops quickly as a result (Figure 4a). Therefore, in the solid particle-rich environment, respirator users are likely to feel the breathing resistance rather quickly. In this case, the build-up of resistance can determine the useful service life of filters. On the contrary, oily aerosols hardly clog the filter media; instead, the liquid spreads on the surface, quickly masking the surface charges of electret filters. Thus, the loading of oily aerosol usually deteriorates the filter performance more quickly than that of solid particles (Figure 5). 

All tested webs had 89%–90% porosity (Table S2 in Supplementary Materials). The pore size distribution for each web is shown in Figure S1 of Supplementary Materials. A lower air resistance of PS_O_2__ ES than PS ES corresponds to the lowered basis weight after the O_2_ plasma treatment, which was probably due to the removal of hydrocarbons in slight amounts, resulting in a creation of large pores (Figure S1). However, the pressure drop difference among the samples was not large except for PP SB. 

From Figure 4a of the NaCl loading, the build-up of resistance was faster for PP MB than PS ES. Faster build-up of resistance for PP MB seems to result from the higher basis weight of PP MB than that of PS ES; the basis weight of the PP MB was nearly two times that of PS ES. In general, the web with larger EFD tends to give lower resistance due to the larger pore sizes, if all other parameters are the same. 

However, in this experiment, the influence of the basis weight on resistance overweighed that of EFD. The resistance during the DOP loading maintained the same, and there was little difference among the samples (Figure 4b).

From Figure 5, the evolvement of penetration with NaCl and DOP loading appeared obviously different. From the NaCl loading (Figure 5a), the penetration of PP MB and PS_O_2__ ES started to decrease immediately upon the particle loading, displaying the mechanical capture mechanism (rather than the electrostatic capture mechanism). On the contrary, PS ES and PS_f_ ES exhibited an increase of penetration in the earlier loading, then the penetration decreased after reaching the maximum penetration. The loading at the maximum penetration can be regarded as the onset of mechanical filtration or the clogging onset, from which the mechanical filtration becomes more dominant than the electrostatic capture mechanism. PS ES and PS_f_ ES showed the clogging onset a little later (5–10 mg of loading) than PS_O_2__ ES and PP MB. 

The delayed onset of mechanical filtration (clogging onset), or the later reach of maximum penetration, may reflect the presence of strong surface charges in the filter media; that is, the particle penetration may increase when the surface charges of the fiber are masked by the deposition of particles. After reaching the maximum penetration, the pores of the filter media begin to clog, and penetration decreases. If there are no surface charges at all, the increase of penetration would hardly appear. The NaCl penetration curves for PS ES and PS_f_ ES are the typical shape for the electret filter media. 

The static charges of the web surface were measured in Table 4, and the result corroborates the presence of strong static charges on PS ES and PS_f_ ES. It is noteworthy that the absolute values of the static charges were similar for PP MB, PS ES and PS_f_ ES. The PP MB was a corona-charged filter media, where it showed the similar level of static charges as PS ES. However, for PP MB, the mechanical filtration appeared to be more dominant than the electrostatic filtration, probably due to the relatively higher thickness and basis weight of the filter web. With oxygen plasma treatment, the static charges were considerably dissipated, and this affected the filtration performance significantly. However, PECVD with fluorine compounds created negative charges on the electrospun surface. The presence of strong static charges on PS ES, PS_f_ ES and PP MB was advantageous for filtering NaCl particles and DOP aerosol with the continued loading (Figure 5 and Table 5). 

**Figure 6:**
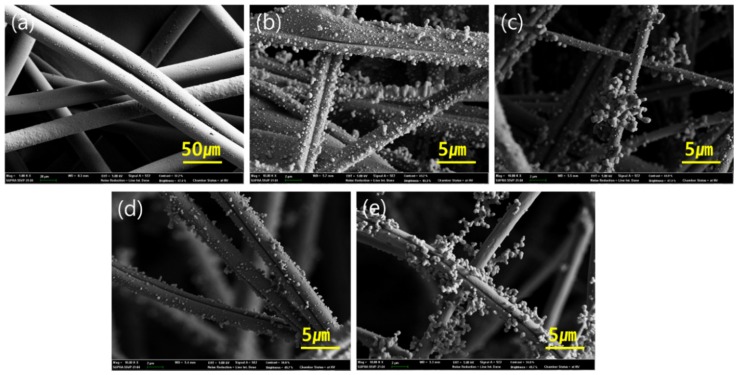
SEM images of nonwoven media after 20 mg of NaCl loading. (**a**) Polypropylene (PP) SB; (**b**) PP MB; (**c**) Polystyrene (PS) ES; (**d**) PS_O_2__ ES; (**e**) PS_f_ ES.

The effect of plasma treatments on PS ES appeared differently, depending on the resulting functionality. The charges of the electrospun web were lost with the O_2_ plasma process, leading to a significant deterioration of filtration performance. From SEM images in Figure 6d, there was little observable physical damage of the PS_O_2__ ES fibers, and the deteriorated filtering performance would be mainly due to the loss of static charges. It can be inferred that the oxygen species on the surface increased the relative permittivity, facilitating the charge decay in the ambient condition [51]. 

To examine the particle loading behavior, SEM images of webs after 20 mg of NaCl loading were observed in Figure 6. On PS_O_2__ ES, particles were adhered directly onto the surfaces instead of forming particle dendrites. On the contrary, PS ES and PS_f_ ES showed dendrite formation, where NaCl particles were adhered onto other layers of the particles, probably by the electrostatic attraction. The dendrite formation was most obvious for PS_f_ ES and PS ES, where the high level of static charges was measured. As the PECVD process was conducted in an electric field, charges can be additionally imposed to the PS_f_ ES. Also, due to the lower surface energy and the relative permittivity of fluorinated compounds, the charge decay of PS_f_ ES could be deterred. However, the overall NaCl penetration of PS_f_ ES was higher than that of PS ES. It is suspected that particle capture mechanisms other than electrostatic attraction may be deteriorated by the surface fluorination, and the adhesion force between the NaCl particles and the surface may be decreased by the lowered surface energy of the fibers. It is worth noting that the mechanical strength of the electrospun web was sufficient to endure the particle and aerosol loading during the test. Supplementary Figure S3 shows the electrospun web before and after NaCl loading, and there was no observable damage after the particle loading.

For DOP loading (Figure 5b,d), PS_f_ ES showed the highest performance in general. The improved efficiency is well attributed to the lowered surface energy of PS_f_ ES, which prevented the quick spreading of DOP on the charged surface, as shown by the lowered DOP CA (Table 2). The delayed masking of surface charges contributed to the improved filtration efficiency against the oily aerosol. Thus, the combined effect of lowered DOP wetting and high static charges for PS_f_ ES appeared to be advantageous for DOP filtration with the continued loading. While the instantaneous DOP penetration was lower for PS ES than for PS_f_ ES, the penetration through PS ES increased very quickly with the continued loading of DOP. The rate of penetration increase was lower for PP MB than PS ES, due to the higher thickness of MB (0.32 mm thickness) than PS ES (0.13 mm). 

For PS ES, DOP penetration increased very quickly once the thin fiber layer was saturated with liquid. The oil spreading on the fiber surface was not clearly observed from SEM images (Supplemental Information Figure S2 in Supplementary Materials). The important parameters of the filtration performance are summarized in Table 5. 

For filter media that is used for particulate respirators, low penetration and low resistance are preferable, as they can be interpreted as high protection and good breathing comfort for users. As penetration and resistance are compensating factors, a concept of quality factor (QF) is employed to account for both factors simultaneously (Equation 5). The QF determines the inherent quality of the filter media to some extent, and a higher QF is translated to have a higher efficiency at the same resistance. From Figure 5c, PS ES showed the highest QF during the earlier loading of NaCl. The QFs of all filter media converged to a similar level as the clogging of the filter became the main contributor to this QF. While PS_f_ ES showed lower efficiency than PS ES, the high level of static charges on PS_f_ ES helped in effectively attracting NaCl particles, forming dendrites. The result demonstrates that the electrospun media can be applied as an effective filter media for N-type class filters.

From Figure 5d, the QF against DOP was highest for PS_f_ ES until about 50 mg of DOP loading. Again, the QF converged with the extreme load of DOP, as the filter media lost most of its performance. The result showed that the combined effects of a lowered surface energy and a high level of static charges for PS_f_ ES fibers were advantageous for the filtration of oily liquid, and this type of media would be relevant for R- or P-type filter application. 

In general, it is more challenging to keep the performance against oily mist, due to the quick masking of surface charges during the oil loading. Based on the results, the PECVD process is suggested as a facile and effective method to design the filter media with superior filtration performance especially for liquid aerosol.

## 4. Conclusions

In search of a facile design strategy for an effective filter media, the study investigated the effect of plasma-based surface modification on the charge holding capability and the resulting filtration performance. PS electrospun web (ES) had inherent static charges of ~3.7 kV due to its electric field-driven process. Compared to the PP MB electret, PS ES showed higher filtration performance in terms of quality factor (QF), even with thinner and less material. When oxygen species were created on the PS surface by the O_2_ plasma process, the static charges were lost considerably as the polarity of PS_O_2__ ES was increased, and this led to the deteriorated filter performance. On the contrary, PECVD with C_4_F_8_ created the negative static charges on the web surface to ~−3.9 kV. Additionally, the fluorinated surface decreased the surface energy, lowering the wettability of the oily liquid. The combined effect of lowered DOP wettability and the high static charges contributed to the enhanced filtration performance of PS_f_ ES against the DOP aerosol. However, its performance against NaCl particles was lowered to some extent, probably due to the lowered adhesion of NaCl on the fluorinated surface. Particles on the charged media such as PS ES and PS_f_ ES formed dendrites, as particles were attracted to other layers of particles by the electrostatic interaction. Based on the findings, the PECVD process is suggested as a facile fabrication method to design effective filter media, especially for liquid aerosol. Though further questions need to be answered on the effect of lowered surface energy on particle adhesion, the results of this study will broadly impact the design strategy of media for the superior filtration of oily aerosol with the enhanced service life.

## Figures and Tables

**Table 1:** Characteristics of webs.

Web	BW (g/m^2^)	Thickness (mm)	EFD (μm)	Measured Dia. (μm)
PP SB	23 (±1)	0.23 (±0.04)	25.0	23.0 (±1.5)
PP MB	30 (±2)	0.32 (±0.07)	5.1	4.1 (±1.5)
PS ES	14 (±1)	0.13 (±0.03)	3.7	2.2 (±1.1)
PS_f_ ES	13 (±3)	0.13 (±0.02)	3.5	2.5 (±1.3)
PS_O_2__ ES	10 (±1)	0.10 (±0.03)	3.3	3.1 (±1.5)

**Table 2:** Surface wettability of different webs.

CA or ShA	PS ES	PS_O_2__ ES	PS_f_ ES	PP SB	PP MB
WA CA (°)	157° (±1)	0°	159° (±4)	154° (±3)	155° (±2)
WA ShA (°)	41° (±1)	NA	11° (±2)	34° (±2)	14° (±1)
DOP CA (°)	0° (NA)	0° (NA)	149° (±2)	0° (NA)	0° (NA)

Note: The CA of dioctyl phthalate (DOP) was measured in 5 s of deposition, because the DOP droplet spread slowly until 5 sec after deposition. The DOP CA became stable in 5 s.

**Table 3:** X-ray photoelectron spectroscopy (XPS) atomic concentration (%) of surfaces.

Specimen	C (%)	O (%)	F (%)
PS	100	-	-
PS_O_2__	84.71	15.29	-
PS_f_	70.87	-	29.13
PP	100	-	-

**Table 4:** Static charges of nonwoven media.

PP MB	PP SB	PS ES	PS_O_2__ ES	PS_f_ ES
−3.5 kV (±0.7)	−0.5 kV (±0.1)	3.7 kV (±0.5)	1.2 kV (±0.2)	−3.9 kV (±0.5)

**Table 5:** Summary of the filtration performance.

Title	PS ES	PS_O_2__ ES	PS_f_ ES	PP MB
Initial resistance (mmH_2_O)	5.4 (±0.3)	4.8 (±0.6)	5.3 (±0.6)	6.6 (±0.6)
NaCl initial Pn (%)	13.0 (±1.3)	68.6 (±7.0)	27.3 (±3.4)	28.7 (±2.1)
NaCl max. Pn (%)	17.8 (±1.4)	68.9 (±7.0)	30.0 (±2.9)	29.4 (±2.7)
Challenge load of NaCl at max. Pn (%)	8.8 (±1.7)	0.5 (±0)	5.8 (±0.5)	1.1 (±0)
DOP initial Pn (%)	29.9 (±6.2)	57.6 (±4.1)	37.1 (±3.3)	37.6 (±1.8)
DOP max Pn at 200 mg load (%)	77.0 (±2.5)	75.3 (±2.7)	73.0 (±1.7)	75.8 (±1.7)

Note: Initial resistance is the mean value of resistance measured from the DOP and NaCl tests.

## Supplementary Materials

The following are available online at https://www.mdpi.com/2073-4360/11/9/1490/s1, Figure S1: Pore size distribution of webs; Figure S2: SEM images of different webs before and after 40 mg of DOP; Figure S3: Electrospun filter web images before (a) and after (b) NaCl particle loading; Table S1: Reference values of surface energy components of liquids; Table S2: Solidity and porosity of different webs.
