# Erratum: Facile Functionalization via Plasma-Enhanced Chemical Vapor Deposition for the Effective Filtration of Oily Aerosol. *Polymers* 2019, *11*, 1490

**DOI:** 10.3390/polym12061422

**Published:** 2020-06-25

**Authors:** Sanghyun Roh, Sungmin Kim, Jooyoun Kim

**Affiliations:** 1 Department of Textiles, Merchandising and Fashion Design, Seoul National University, Seoul 08826, Korea; shtkdgus011@snu.ac.kr (S.R.); sungmin0922@snu.ac.kr (S.K.); 2 Research Institute of Human Ecology, Seoul National University, Seoul 08826, Korea

The authors wish to make a change to the published paper [1]. In the original manuscript, there is a mistake in Equation (5). The corrected Equation (5) is presented below.

(5) $$\mathrm{Quality}\;\mathrm{factor}\;(\mathrm{mm}\;H_{2}O^{-1})\;=\;\frac{-\ln(\%\;\mathrm{penetration}/100\%)}{\mathrm{pressure}\;\mathrm{drop}\;\left(\mathrm{mm}\;H_{2}O\right)}$$

The authors apologize for any inconvenience caused and the change does not affect the scientific results. The manuscript will be updated, and the original will remain online on the article webpage at https://www.mdpi.com/2073-4360/11/9/1490.

## References

[1] Roh S., Kim S., Kim J. (2019). Facile Functionalization via Plasma-Enhanced Chemical Vapor Deposition for the Effective Filtration of Oily Aerosol. Polymers.

