# Supplementary material for: Facile Functionalization via Plasma-Enhanced Chemical Vapor Deposition for the Effective Filtration of Oily Aerosol
Source: Polymers (Basel). 2019 Sep 12;11(9):1490. doi: 10.3390/polym11091490 (PMC6780184; doi:10.3390/polym11091490)
Supplement: Supplementary file 1 [file polymers-11-01490-s001.pdf]

Facile functionalization via plasma enhanced chemical vapor deposition for effective filtration of oily aerosol

Sanghyun Roh[1], Sungmin Kim[1,2], Jooyoun Kim[1, 2] *

[1]Department of Textiles, Merchandising and Fashion Design, Seoul National University, Seoul 08826, Republic of Korea

[2]Research Institute of Human Ecology, Seoul National University, Seoul 08826, Republic of Korea

E-mail: *jkim256@snu.ac.kr

Tel.: +82-2-880-6846

Table S1. Reference values of surface energy components of liquids

Liquid	γ_L (mN/m)	γ_L^d (mN/m)	γ_L^p (mN/m)	P_L (Polar Ratio)
Water (WA)	72.8	21.8	51.0	0.70
Methylene iodide (MI)	50.8	50.4	0.40	0.008

Note: γ_L, overall surface energy of liquid; γ_L^d, dispersive component surface energy of liquid; γ_L^p, polar component surface energy of liquid; P_L, ratio of the polar component to the overall surface energy of liquid.

$$\gamma_{SL} = \gamma_S + \gamma_L - 2\sqrt{\gamma_S^d \cdot \gamma_L^d} - 2\sqrt{\gamma_S^p \cdot \gamma_L^p} \quad \text{(Eq. S1)}$$

$$\gamma_S = \gamma_{SL} + \gamma_L \cos\theta \quad \text{(Eq. S2)}$$

$$\gamma_L(1 + cos\theta) = 2\sqrt{\gamma_S^d \cdot \gamma_L^d} - 2\sqrt{\gamma_S^p \cdot \gamma_L^p} \quad \text{(Eq. S3)}$$

$$\gamma_S = \gamma_S^d + \gamma_S^p \quad \text{(Eq. S4)}$$

θ: contact angle of liquid on solid surface

γ_{SL}: interfacial energy between solid and liquid

γ_S: surface energy of solid

γ_S^d: dispersive component surface energy of solid

γ_S^p: polar component surface energy of solid

γ_L: surface energy of liquid

γ_L^d: dispersive component surface energy of liquid

γ_L^p: polar component surface energy of liquid

Table S2. Solidity and porosity of different webs

Web	Solidity	Porosity (%)
PP SB	0.11	89
PP MB	0.10	90
PS ES	0.11	89
PS_f ES	0.10	90
PS_{O2} ES	0.10	90

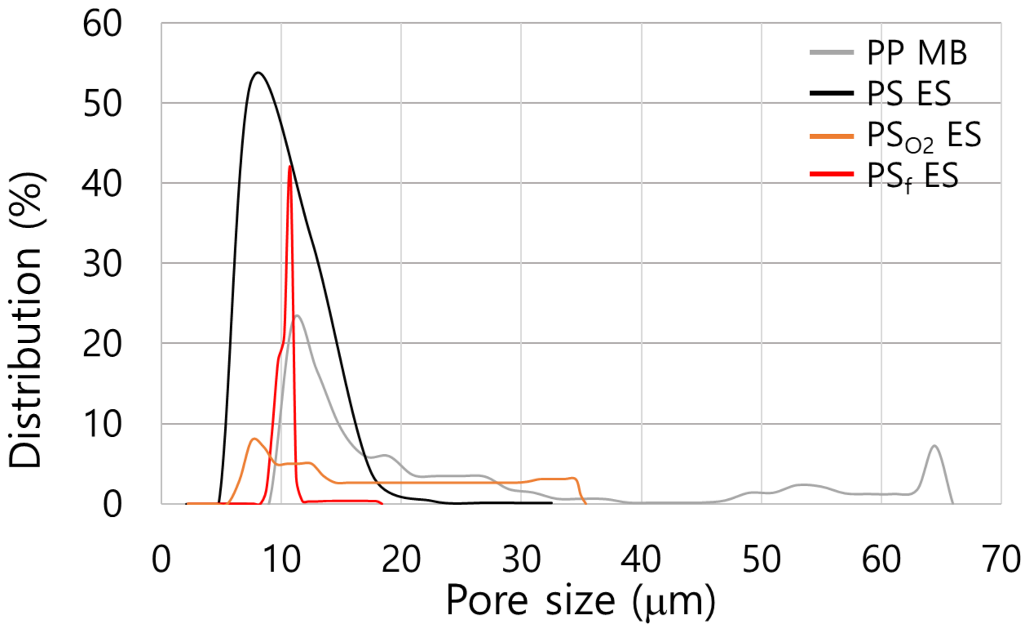

Figure S1. Pore size distribution of webs.

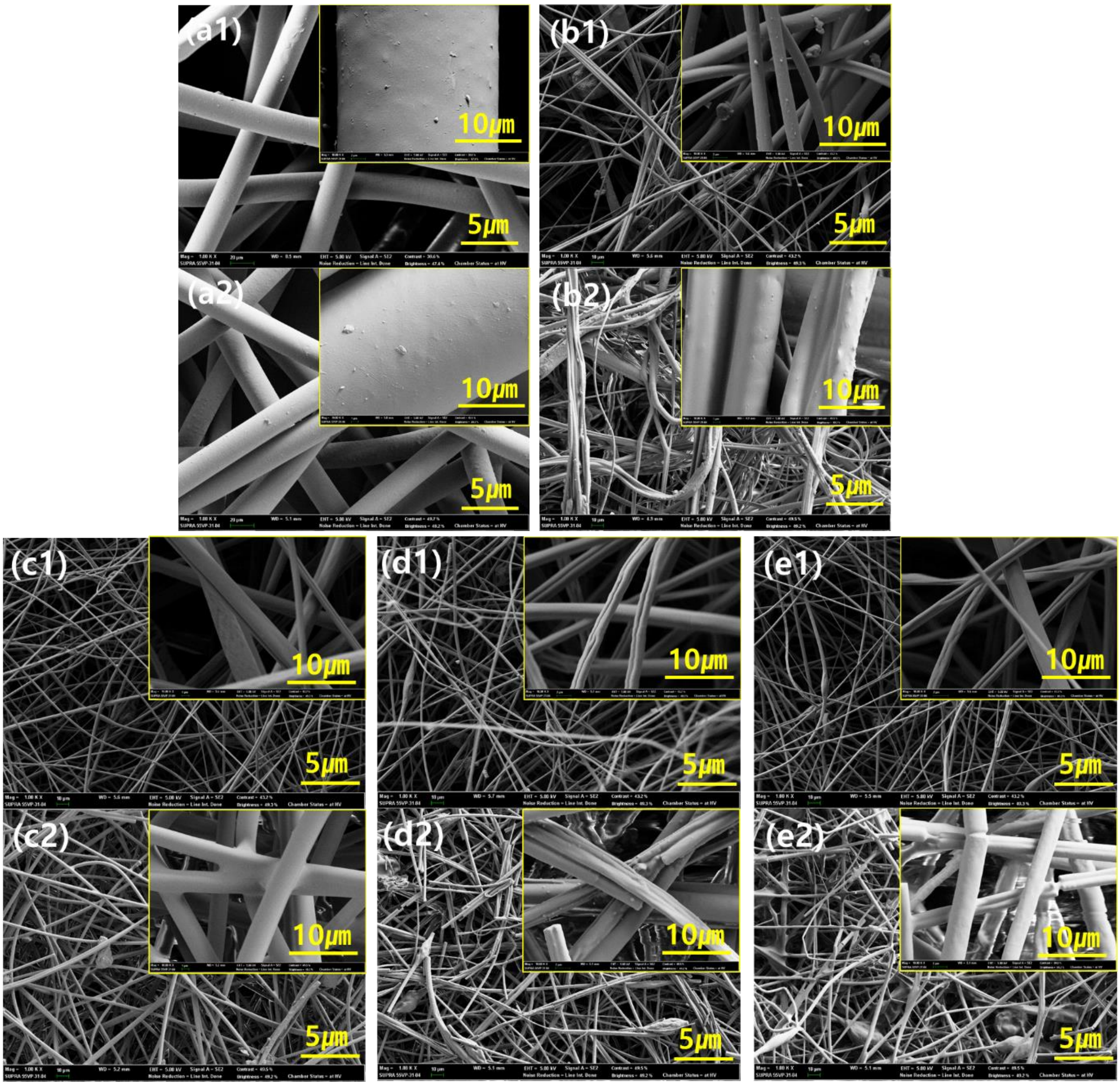

Figure S2. SEM images of different webs before and after 40 mg of DOP loading. (a1) PP SB; (a2) PP SB with DOP; (b1) PP MB; (b2) PP MB with DOP; (c1) PS ES; (c2) PS ES with DOP; (d1) PS_{O2} ES; (d2) PS_{O2} ES with DOP; (e1) PS_f ES (e2) PS_f ES with DOP.

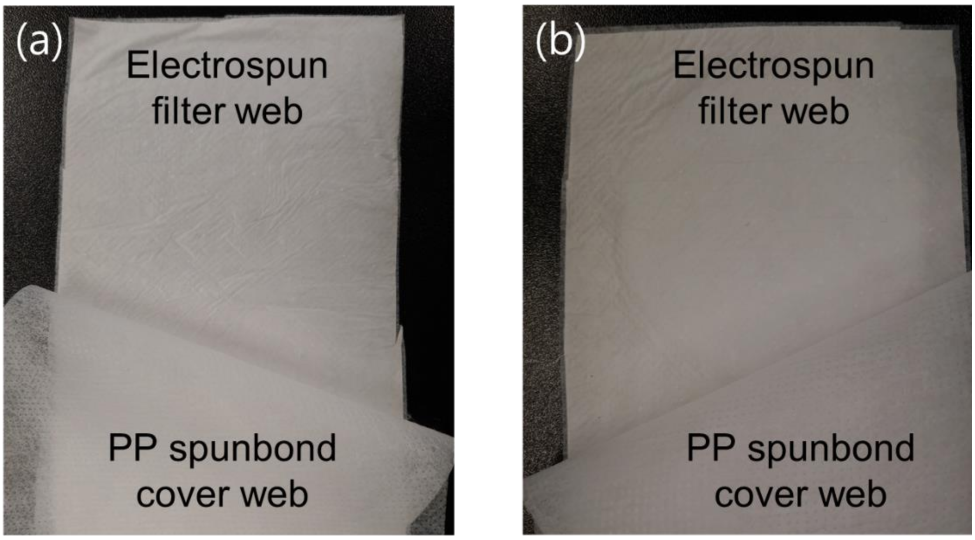

Figure S3. Electrospun filter web images before (a) and after (b) NaCl particle loading.
